# Cellular damage and apoptosis along with changes in NF-kappa B expression were induced with contrast agent enhanced ultrasound in gastric cancer cells and hepatoma cells

**DOI:** 10.1186/1475-2867-12-8

**Published:** 2012-03-15

**Authors:** Zao Jiang, Wei Wu, Meng-lu Qian

**Affiliations:** 1 Department of Oncology, Zhong-Da Hospital & Clinical medical college, Southeast University, 87# Ding-Jia-Qiao Road, Nanjing 210009, China; 2 Key Laboratory of Environmental Medicine and Engineering, Minister of Education, Public health college, Southeast University, Nanjing, China; 3 Acoustics institution, Tongji University, 200092 Shanghai, China; 4 Department of Oncology, Zhongda Hospital, Southeast University/Key Laboratory of Environmental Medicine and Engineering, Minister of Education, Public health college, Southeast University, 87# Ding-Jia-Qiao Road, Nanjing 210009, China

**Keywords:** Ultrasound, Contrast agent, Apoptosis, NF-kappa B, Cancer cell

## Abstract

**Background:**

The effect of cell injury and apoptosis induced by ultrasound with contrast agent has been verified. Contrast agent enhanced apoptosis and expression of genes that related to apoptosis and are responsive to ultrasound. This effect was associated with reactive oxygen species (ROS) production induced by the sonochemical reaction, as reported in previous studies. NF-kappa B may be one of the factors involved in oxidizing reactions or modulation during the process of ultrasound inducing apoptosis.

**Results:**

Ultrasound irradiated gastric cancer cells (SGC7901 cell line) and hepatocellular carcinoma cells (SMMC-771 cell line) cultured in medium containing contrast agent. Significant cellular damage and apoptosis were observed in the bath cells incubated for 24 hours following 120 seconds ultrasonic irradiation. I kappa B alfa expression synchronously increased in the treatment groups of both the cell lines, and the down-regulated expression of NF-kappa B influenced its-regulated expression of genes that related to apoptosis. Production of intracellular ROS and elevation of NF-kappa B level occurred after incubation of the cells for 1 hour following ultrasonic treatment.

**Conclusions:**

Our result suggested that contrast agent enhanced the biological effect of ultrasound. Their reaction might stimulate the transitory expression of NF-kappaB, and subsequent elevation in IκBalfa expression could lead to the apoptosis of SGC7901 cells and SMMC-771 cells.

## Background

Studies regarding the ultrasonic effects on the proliferation of cells have gradually led to the development of a new field of ultrasonic biological research and promoted the emergence of a new direction for ultrasonic medical therapy [1]. Furthermore, low-frequency ultrasonic (20 khz-2 Mhz) irradiation has the ability to directly kill tumor cells including inhibiting tumor cell proliferation and inducing apoptosis [2,3]. The mechanism of inhibition of tumor cell growth may be related to the cavitation effect of low-frequency ultrasound, because free radicals, produced via the cavitation effect, interrupt the replication of normal double-stranded deoxyribonucleic acid (DNA) by promoting the polymerization of DNA strand and restrain cell growth [4]. The biomechanics study regarding the dynamic characteristics of an ultrasound contrast agent suggested that ultrasound irradiation with a microbubble contrast agent may cause cellular injury[5]. In combination with a microbubble contrast agent, ultrasound might promote cellular injury, including the initiation of apoptosis and cell injuries through ultrasonic cavitation effects or via other approaches [6-8].

In previous studies, free radicals were considered to be the main products of ultrasonic irradiation with microbubble agent and as an important factor responsible for cellular injury caused by ultrasound treatment [9,10]. Free radicals, including reactive oxygen species (ROS), can also activate some cellular factors such as nuclear factor kappa B (NF-kappa B); NF-kappa B is one of the cellular factors primarily involved in oxidizing reactions or in inducing "oxidative stress"[11,12]. Although NF-kappa B is known to modulate the signaling pathways responsible for cellular proliferation and degeneration, its activity is regulated by the binding of a cytoplasmic inhibitor protein, I kappa B [13-15]. However, such alterations in the levels of these factors have been not reported when cells are subjected to ultrasound irradiation with contrast agent.

In order to determine the biological effects and changes in the levels of relevant cellular factors and to elucidate the possible mechanism underlying the effects of low-frequency ultrasound combined with microbubble agents, we used a contrast agent Levovist to determine the changes in the expressions of NF-kappa B and I kappa B in cultured gastric cancer cells and hepatocellular carcinoma cells subjected to low-frequency ultrasound irradiation.

## Results

### Test of inhibiting cells proliferation

The cells were incubated for 24 hours after ultrasonic irradiation for 60, 90, 120 and 150 seconds, the lowest cell survival rates were observed in the cells irradiated for 120 seconds compared with those of the control group. The survival rate of the SMMC-7721 cells was 51.52 ± 3.51% and that of the SGC-7901 cells was 49.63 ± 4.21%. Similar results were obtained when the cells were irradiated for 120 seconds with ultrasound and contrast agent after incubation for 1, 24 and 48 hours.

### Ultrasound-induced changes in the intracellular levels of reactive oxygen species (ROS) and superoxide dismutase (SOD) in the medium containing the contrast agent

Ultrasound irradiation of the cells cultured in the medium containing contrast agent led to the production of ROS and abatement of SOD activity. Significant effects were observed at 1 hour incubation after 120 seconds of ultrasonic exposure. In SGC7901 cells, Changes in the intracellular levels of ROS were significantly (81.40 ± 1.31 for group D *vs*. 23.20 ± 1.22 for group A after incubation for 60 min, *p *< 0.05) and SOD(69.52 ± 2.81 U/ml for group D *vs*. 165.06 ± 1.14 U/ml for group A after incubation for 60 min, *p *< 0.05). Similar changes occurred in SMMC-7721 cells after incubation for 1 hour. The intracellular level of ROS was increased (77.40 ± 1.1 for group D *vs*. 23.20 ± 1.2 for group A, *p *< 0.05), and the level of SOD was decreased (58.87 ± 1.91 for group D *vs*. 149.15 ± 5.72 U/mL for group A, *p *< 0.05.).

### Morphological changes of cellular damage and apoptosis

**Figure 1:**
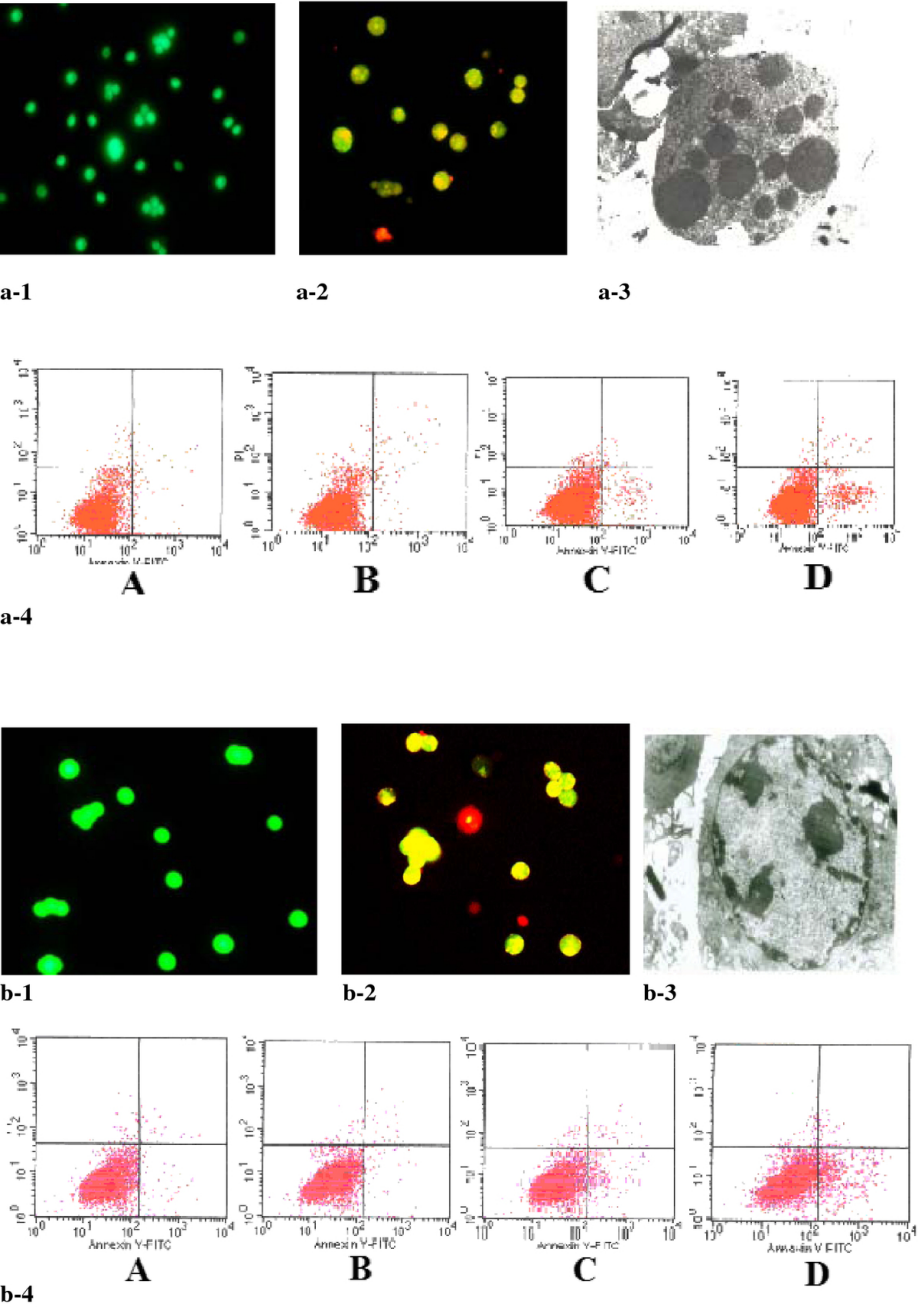
**Morphological changes of the apoptosis in cancer cells were observed by AO/EB double fluorescent staining under fluorescent microscope (control groups in Figure 1a-1 and Figure 1b-1, group D in Figure 1-a2 and Figure 1-b2.), transmission electron microscope(group D in Figure 1-a3 and Figure 1-b3) and flow cytometry(Figure 1a-4 and Figure 1b-4)**. (SGC-7901 cells were in Figure 1a, SMMC-7721 cells in Figure 1b.).

Both SGC7901 and SMMC-7721 cells showed different kinds of changes in cellular morphology after treatment with ultrasound combined with microbubble agent. The main morphological changes observed in the cells were as follows: mitochondria swelling or vacuolation with disappearance of mitochondria cristae, cytoplasmic rarefaction, formation of vacuoles of various sizes, reduced electron density matrix, and dilation of endoplasmic reticulum. Apoptosis was also observed in some cells showing characteristic apoptotic features of cell shrinkage and cytoplasmic condensation, blistering of cytoplasm, formation of apoptotic bodies, nuclear condensation, and assembly of nuclear chromatin toward the inner surface forming clumps or crescent-shaped bolus. As demonstrated, treatment by ultrasound with contrast agent induced typical apoptotic morphological changes, including orange cell shrinkage with condensation and fragmentation of nuclei (Figure 1).

### Flow cytometry-based confirmation of apoptosis in cells after treatment with both ultrasound and contrast agent

The apoptosis rates in the SMMC-7721 cells treated with ultrasound and microbubble agent were significantly higher (27.31 ± 4.14) than those in group A (1.69 ± 0.27, *P *< 0.05), group B (2.51 ± 0.32,), and group C (15.24 ± 2.16). Similar results were observed in the SGC7901 cells. The apoptotic rates in SGC7901 cells in group D (21.15 ± 4. 68) were significantly lesser than those in group A (1.15 ± 0.37 *P *< 0.05), group B (2.39 ± 0.65,), and group C (11.7 ± 2.68) (Figure 1).

### Western blot analysis showed the transient expression of NF-kappa B in the cells treated with ultrasound and contrast agent

**Figure 2:**
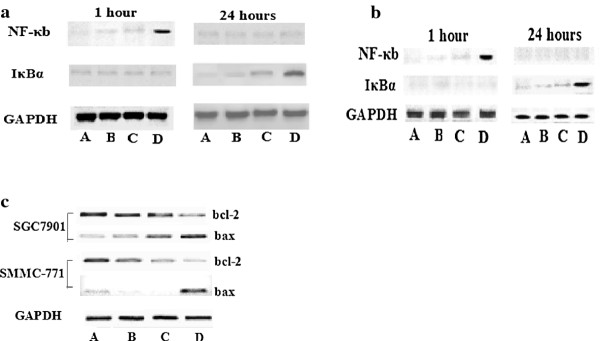
**The expressions of NF-κB and IκBα in SCG7901 cells (Figuer 2a) and SMMC-7721 cells (Figure 2b) were after incubation at different times following ultrasound irradiation for 120 second after incubation for 1 hour or 24 hours**. Expressions of genes (Figure 2c) that related to apoptosis regulated by NF-kappa B were after irradiation for 24 hours.

Similar results were obtained in both SMMC-7721cells and SGC7901 cells. NF-kappa B expression levels in both cells were remarkably higher than that of control group when the cells were incubated for 1 hour after 120 seconds treatment with ultrasound and contrast agent. At the same time, I kappa B-alfa expressed indistinctly. However, enhanced expression of I kappa B-alfa was observed when the cells were incubated for 24 hours after the treatment. Meanwhile decreased expressions of NF-kappa B and bcl-2 were observed respectively in the both cell lines, and reciprocal expression of bax also emerged in those cells (Figure 2).

## Discussion

Ultrasonic microbubble contrast agent might produce biological effects on cells, because such agents are able to induce cellular and cell membrane injuries [1,6]. ROS induced by ultrasound irradiation are thought be responsible for the biological effects of ultrasound irradiation [16,17]. Ultrasonic microbubble contrast agents have the ability to produce oxyradicals, which has been confirmed by spin resonancespectroscopy [18]. These radicals might induce apoptosis and necrosis in tumor cells via several targets, such as cell membranes, intracellular bioactive molecules, and DNA [19]. In this study, we found that low-frequency ultrasonic irradiation with microbubble agent promoted apoptosis and inhibited cellular proliferation in tumor cells and vascular endothelial cell. This effect was associated with the attenuation of the SOD activity in the culture medium. Consumption of active oxygen scavengers indicates an increase in the level of ROS produced during cellular damage caused by treatment with ultrasound and microbubble agent. This phenomenon has been assessed by other researchers who have suggested a sonochemical mechanism underlying this effect [20].

Apoptosis can be induced by treating cells with ultrasound and contrast agent. ROS-mediated injury may be one of the important factors responsible for the biological effects of ultrasound [17,18]. Increase in the expression of NF-kappa B and I kappa B-alfa has been often associated with the induction of apoptosis [14]. These nuclear factors might be related with the sequential induction of cell death caused by ultrasound.

ROS can activate the expression of intracellular NF-kappa B. This ubiquitous cytokine is involved in the regulation of various cellular effectors molecules and is present in an inactive form as a dimer with I kappa B-alfa, in the absence of any stimulation. The activity of NF-kappa B proteins is regulated by I kappa B-alfa proteins [21]. When cells are stimulated, NF-kappa B is activated and is translocated to the nucleus, where it binds to the target gene promoter and activates its transcription [15]. A variety of extracellular signals can stimulate the activation of NF-kappa B, such as ROS, ultraviolet irradiation, double-stranded RNA, cytokine interleukin-1, tumor necrosis factor-α, lipopolysaccharide, and viruses [11-13]. Because the regulation by NF-kappa B activation, de novo I kappa B-alfa can reintegrate with NF-kappa B to form a dimeric compound to inhibit the activation of NF-kappa B. Although I kappa B-alfa is synthesized de novo, NF-kappa B activity remains sustainable for several hours [22]. We found that the protein level of NF-kappa B was markedly augmented at 1 hour after treatment with ultrasound irradiation and microbubble agent, suggesting that the cellular damage was associated with the oxidation induced by ultrasound and microbubbles. Hence, the biological effects of ultrasound were speculated to be associated with the ultrasound chemical reaction. At 24 hours after ultrasound treatment, the enhanced expression of I kappa B suggested that emerge of the activities of intracellular biological mediation produced through ultrasound chemical reactions, corresponding with the general regulation of modulating cellular proliferation in this type of cytokines [21,22]. We found that I kappa B-alfa expression promoted apoptosis in the experimental groups. Ultrasonic irradiation increased the level of ROS and induced the expression of NF-kappa B and I kappa B in succession. This finding suggests that the biological effect of sonochemistry is one of related factors in ultrasound killing cells with microbubble agent. Due to the dynamic characteristics of ultrasound contrast agents [5], ultrasound microbubble agents undergo reactions involved in ultrasound physics and ultrasound chemistry, damaging cell membranes and intracellular structures, and ultimately resulting in cellular injury or even cell death [23].

## Conclusion

In this study, SMMC-771 tumor cells and SGC7901 cells were injured or induced apoptosis after treatment with low-frequency ultrasound with contrast agent. The apoptotic mechanism might be related to transitory expression of NF-kappa B stimulated by enhanced ultrasonic effect of contrast agent, and subsequent elevation in I kappa B alfa expression leads to the apoptosis and alterations of related apoptotic genes of cancer cells. The study results may provide experimental evidence or basis for using ultrasonic contrast agents to investigate their use in the treatment of cancer.

## Methods

### Cells and culture medium

Gastric cancer SGC7901 cell line and SMMC-7721 hepatoma cell line (Cell bank, Shanghai, China) were digested with 0.25% trypsin, and a single cell suspension was obtained; the cells were cultured in RPMI 1640 medium (GIBCO, USA) containing 10% fetal calf serum (Sijiqing, Hangzhou, China), 100 U/mL of penicillin, and 100 mg/mL of streptomycin at 37°C in a 5% CO_2 _incubator. For all experiments, a cell density of 2 × 10^5^/mL was used.

### Ultrasonic apparatus and contrast agents

An ultrasonic surgical device (Jiangsu Meidakang, Nanjing, China) was used as an ultrasound apparatus, as described previously [24]. Before every experiment, the apparatus was adjusted using an Ultrasound power meter (Model UPM-DT-1; Ohnic Instruments Co., Maryland, Swiss). Experimental irradiation power was adjusted to 0.5 W on the reading meter, and an effective output value was adjusted to about 0.159 W/cm^2^; this value was determined by measuring the outputs at 1 W, 0.5 W, and 0.25 W, according to the results of a preliminary experiment with the apparatus[25]. Contrast agent, Levovist (SHU 508A, Schering, Berlin, Germany) was added in 200 mg/ml into culture medium before ultrasound irradiation [8].

### Experimental design

Two cell lines (SGC7901 and SMMC-7721) were divided into the following groups: a control group (A), without any interventions; simple microbubble group (B) that was treated with a simple microbubble agent alone; simple ultrasound group (C) that was treated with ultrasound alone; and ultrasound combined with microbubble agent group (D). The groups that received ultrasonic irradiation were further divided into 4 subgroups according to the ultrasonic irradiation time of 60, 90, 120, and 150 seconds. The cells treated with ultrasonic irradiation were placed in a latex finger glove. The glove was suspended under degassed water in a constant temperature bath, maintained at 37°C and was made to be in contact with a transducer placed inside the bucket. Immediately before exposing the cells to ultrasound, we added the microbubble contrast agent at a concentration of 1:3. After the treatment, the conditioned medium was replaced with a fresh one. For viability testing, 200 μl of the sample was used, and the remaining sample was incubated for 1, 24 and 48 hours under the same conditions until further analysis.

### Measurement of cell survival rate

Cell survival rate was measured using the methyl thiazolyl tetrazolium (MTT) assay kit (Sigma, USA). Absorption (A570) was measured at 570 nm, and cell survival rate was calculated using the following formula: cell survival rate = [optical density value (experiment group)/optical density value (control group)] **× **100%.

### Measurement of reactive oxygen species (ROS) and superoxide dismutase (SOD) levels

The level of intracellular ROS was evaluated with a fluorescent probe, 2',7'-dichlorofluorescin diacetate (DCFH-DA) in reactive oxygen species assay kit (Applygen Technologies Inc. Beijing, China) and a FACS Calibur (BD Pharmingen)[26]. The activities of superoxide dismutase (SOD) were assayed by the kits (Jiancheng Biotechnology Institute, Nanjing, China).

### Morphological survey

The cells were observed under optics inverted microscope (OLYMPUS-CK2; Olympus, Japan) for routine morphological investigation. After treatment with the agents, the cells were also observed under a transmission electron microscope (Hitachi-660, Hitachi, Japan) after centrifugation at 1000 rpm for 5 min, 2 times each. Apoptotic morphological changes in the cells were detected by staining with AO/EB. Briefly, 1 ml of a stock solution containing 100 mg/ml each of AO and EB was added to 25 ml of cell suspension. Cells were examined using fluorescence microscopy. Viable cells were colored green with intact nuclei. Apoptosis was demonstrated by the appearance of orange cell shrinkagewith condensation and fragmentation of nuclei.

### Flow cytometry-mediated detection of apoptosis

After the cells were treated with ultrasound irradiation, they were detected using the calcium-binding protein V-fluorescein isothiocyanate (AnnexinV-FITC) staining kit (Immuno tech Co. France) by using a flow cytometer (Beckman Coulter, USA).

### Western blot

Western blot analysis was performed for detecting the expressions of NF-kappa B, I kappa B alfa, bcl-2 and bax in the cells (SGC7901 cells or SMMC-7721 cells), as described in detail previously [13,22]. Enhanced chemiluminescence (ECL) (Santa Cruz, CA, USA) was used to detect the protein bands, and the results were analyzed using the BioProfil gel electrophoresis image analysis system after gray-scale integration of the electrophoresis bands corrected with glyceraldehyde-3-phosphate dehydrogenase (GAPDH) (Boaosheng, Beijing, China).

### Statistical analysis

The experimental data were presented as mean ± standard error and analyzed using statistical package for social sciences (SPSS) 13. 0 statistical software package. Numerical data were compared using analysis of variance (ANOVA). Statistical significance was set at α = 0.05.

## Abbreviations

DCFH-DA: 2',7'-dichlorofluorescin diacetate; ECL: enhanced chemiluminescence; Bcl2: B cell lymphoma gene 2; GAPDH: glyceraldehyde-3-phosphate dehydrogenase; AO/EB: acridine orange/ethidium bromide; FACS Calibur; fluorescent actived cell sortor or flow cytometer; MTT: methyl thiazolyl tetrazolium; ROS: reactive oxygen species; SOD: superoxide dismutase; NF-kappa B: nuclear factor kappa B.

## Competing interests

The authors declare that they have no competing interests.

## Authors' contributions

JZ performed the morphological survey, experimental design, analyzed the data, wrote and revised the manuscript. WW carried out cell culture, ultrasound treatment, the experiments of Western blotting, fluorescence staining and made the draft of the manuscript as co-first author. QML carried out experimental design of ultrasound principle. All authors read and approved the final manuscript.
